# Long-term outcomes and safety of radical transmediastinal esophagectomy with preoperative docetaxel, cisplatin, and 5-fluorouracil combination chemotherapy for locally advanced squamous cell carcinoma of the thoracic esophagus

**DOI:** 10.1186/s12957-020-02023-2

**Published:** 2020-09-22

**Authors:** Yukinori Yamagata, Kazuyuki Saito, Kosuke Hirano, Masatoshi Oya

**Affiliations:** 1 Department of Surgery, Dokkyo Medical University Saitama Medical Center, 2-1-50, Minami-Koshigaya, Koshigaya City, Saitama Japan; 2 Department of Gastric Surgery, National Cancer Center Hospital, 5-1-1, Tsukiji, Cyuo-ku, Tokyo Japan

**Keywords:** ESCC; Esophageal squamous cell carcinoma, TME; Transmediastinal esophagectomy, NAC; Neoadjuvant chemotherapy, DCF therapy; Docetaxel, cisplatin and 5-fluorouracil combination chemotherapy

## Abstract

**Background:**

It is unknown whether transmediastinal esophagectomy (TME) is an acceptable surgical procedure for locally advanced esophageal squamous cell carcinoma (ESCC). Therefore, we investigated the feasibility of long-term survival after TME with neoadjuvant docetaxel, cisplatin, and 5-fluorouracil combination chemotherapy (DCF therapy).

**Methods:**

This retrospective, observational study included locally advanced resectable ESCC. All patients received two cycles of preoperative DCF therapy (60 mg/m^2^ of docetaxel and cisplatin on day 1 and 700 mg/m^2^/day of 5-FU on days 1–5 in each cycle) followed by radical TME. The main outcomes were survival and the rate of adverse events of chemotherapy and surgery.

**Results:**

Sixteen patients were included in this study. All patients received two cycles of DCF therapy, followed by surgery. The median follow-up duration of the 16 patients was 35.4 months. The 2-year overall survival (OS) was 93.3% (95% confidence interval [CI], 61.3–99.0), and the 3-year OS was 78.8% (95% CI, 47.3–92.7). The 2-year and 3-year relapse-free survivals were both 73.3% (95% CI, 43.6–89.1). Leukopenia and neutropenia occurred in most patients; however, they were controllable. Fifteen patients completed TME, and one was converted to open transthoracic esophagectomy because of tracheal injury. Three-field dissection was performed for 12 of 16 patients (75%), and R0 resection was achieved in 15 of 16 patients (93.8%). Three cases of grade IIIb chylothorax were observed. There was no mortality in this study.

**Conclusion:**

Combined neoadjuvant DCF and TME for locally advanced ESCC was safe and less invasive than traditional therapies and had a satisfactory long-term prognosis.

## Introduction

Esophageal cancer is known to have aggressive metastatic potential with poor prognosis [1]. Additionally, even in relatively early stages of esophageal cancer, lymph node metastases can occur because of rich lymphatic flow in the esophagus [2].

Radical esophagectomy remains the standard treatment for resectable esophageal cancer [1]. To control widespread lymph node metastasis, esophagectomy with cervical to abdominal lymph node dissection, so-called three-field dissection (3FD), has been performed for thoracic esophageal cancer in Japan [3]. However, esophagectomy alone is not sufficient to improve prognosis. Multidisciplinary treatment, i.e., chemotherapy and/or radiotherapy added to surgery, is effective. In Western countries, preoperative neoadjuvant chemoradiotherapy (NACRT) is popular [2].

In contrast, preoperative neoadjuvant chemotherapy (NAC) is the standard therapy in Japan because of the results of the JCOG9907 study [4]. The prognosis of esophageal cancer has improved with multidisciplinary treatment. However, it remains unsatisfactory.

There are two possible ways to further improve the prognosis of esophageal cancer. One is to strengthen preoperative treatment, and the other is to improve the surgical procedure. Combination NAC of 5-fluorouracil (5-FU) and cisplatin (CF therapy) is the standard treatment for locally advanced esophageal cancer in Japan [4]. To strengthen this treatment, we introduced triplet chemotherapy, namely, docetaxel, cisplatin, and 5-FU combination chemotherapy (DCF therapy), which has already been chosen as one of the promising regimen for neoadjuvant therapy in Japan. To improve surgical outcomes, we focused on the approach of esophagectomy. Conventionally, a transthoracic approach to the thoracic esophagus is needed for 3FD. However, transthoracic esophagectomy (TTE) has a high rate of respiratory complications [5], despite thorascopic surgery becoming widespread [6, 7]. Furthermore, the presence of respiratory complications exacerbates the prognosis after esophagectomy [8–10]. Esophagectomy using the transmediastinal approach (non-transthoracic approach) has fewer respiratory complications than TTE [11]. However, radical lymphadenectomy by transmediastinal esophagectomy (TME) is difficult. TME was initially performed as an esophageal extraction [12], and mediastinoscopy was introduced in the 1990s [13, 14]. Mediastinal dissection under inflatable mediastinoscopy was then developed, and complete mediastinal dissection without thoracotomy has become possible in recent years [15, 16].

We first performed TME using an inflatable mediastinoscope in June 2015 and have performed 19 radical esophagectomies for all thoracic or junctional esophageal squamous cell carcinoma (ESCC) and adenocarcinoma cases admitted to our department. In 16 of those 19 cases, which were estimated as clinical stage II to IVa ESCC, we added two cycles of preoperative DCF therapy as NAC. In this report, we evaluated the safety of this therapy and the long-term survival of this cohort.

## Methods

This was a retrospective, observational study. Written informed consent was required from all patients before the start of therapy. We reviewed general information of the patients, histopathological characteristics of the tumors, and data concerning treatment and follow-up. This study was approved by the ethics committee of Dokkyo Medical University Saitama Medical Center (No. 1679).

### Patients

This study included 16 patients with resectable locally advanced ESCC. All patients received two cycles of preoperative DCF therapy, then scheduled radical TME from June 2015 to December 2017. All tumors met the following criteria: (1) histologically proven ESCC, (2) estimated as T1b–T4a and N0–N3 (except T1bN0), (3) estimated as M0, (4) estimated that R0 resection could be available, and (5) estimated that DCF therapy could be tolerable.

### Preoperative chemotherapy

DCF therapy was repeated twice every 4 weeks as preoperative chemotherapy. A dose of 60 mg/m^2^ docetaxel and cisplatin was administered by drip infusion on day 1, and 700 mg/m^2^/day 5-FU was administered by continuous infusion on days 1–5. The dose was appropriately adjusted according to the performance status, renal function, and adverse events (AEs) in the first cycle.

### Surgical procedure

All patients underwent radical TME with regional lymphadenectomy using mediastinoscopy and laparoscopy.

**Fig. 1:**
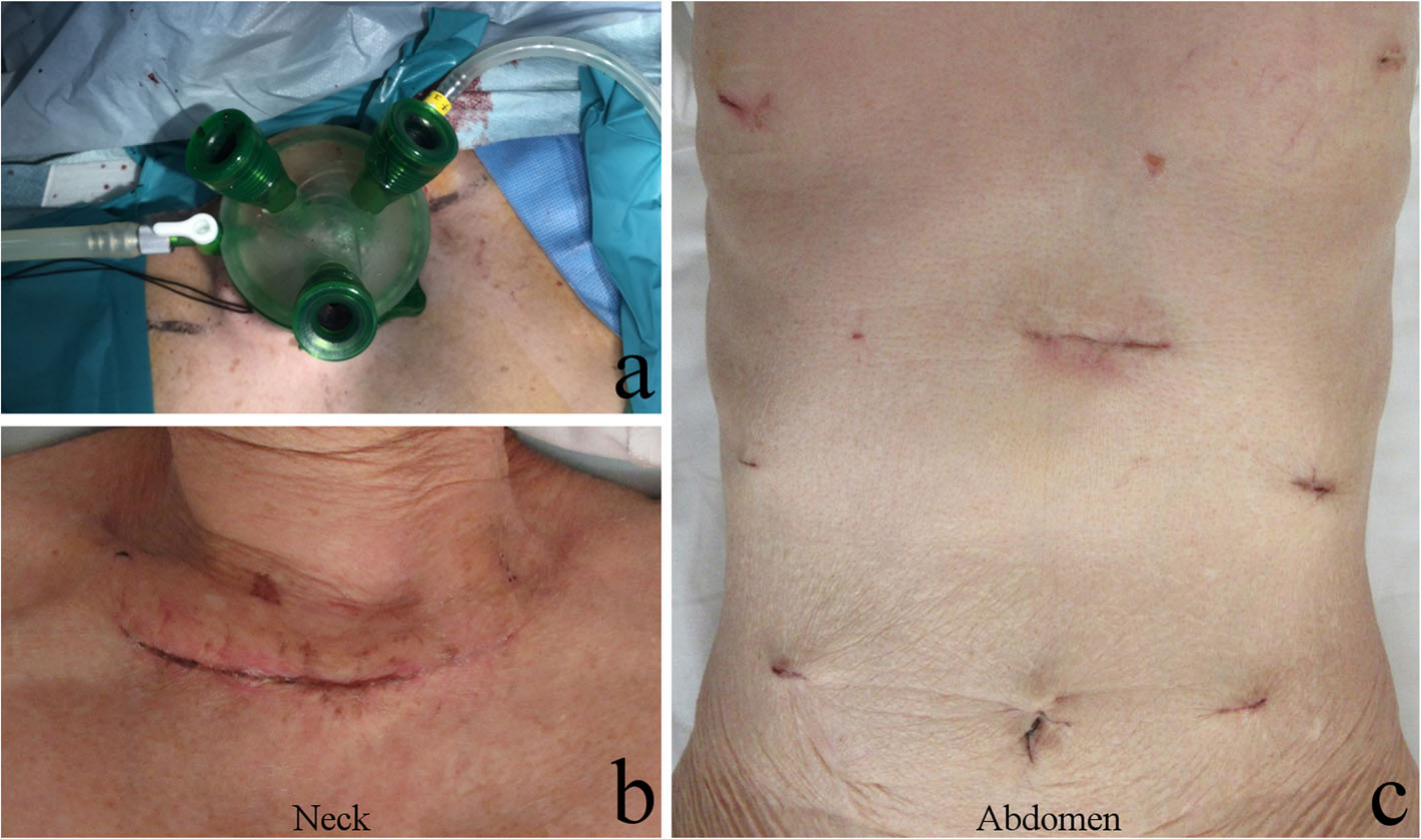
Location of surgical ports. **a** We placed a 4-cm incision on the left neck and inserted a GelPOINT® Mini platform (Applied Medical Co., Ltd, Rancho Santa Margarita, CA, USA), then performed upper to middle mediastinal dissection under inflatable mediastinoscopy. **b** We prepared the stomach tube and dissected around the celiac axis using a laparoscope. **c** We extended the left cervical incision to the right side, and cervical dissection was performed in direct view

**Fig. 2:**
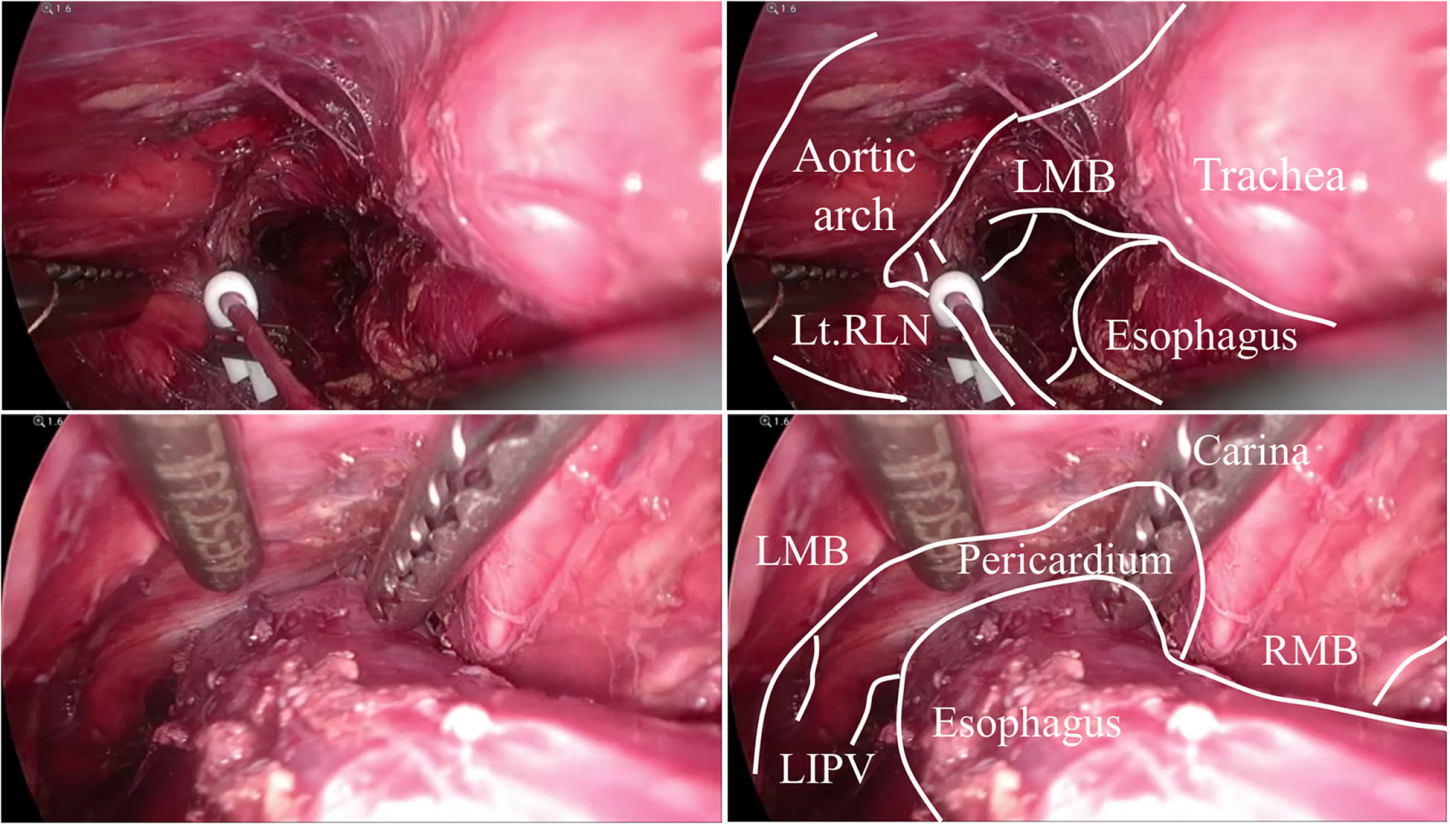
Mediastinoscopic images after mediastinal dissection. Upper: complete dissection of the left recurrent laryngeal nerve and the left tracheobronchial lymph nodes (No.106recL and 106tbL, according to the Japanese Classification of Esophageal Cancer). Lower: complete dissection of the subcarinal and bilateral main bronchus lymph nodes (No. 107 and 109R and 109 L, the same as above). LMB, left main bronchus; Lt. RLN, left recurrent laryngeal nerve; RMB, right main bronchus; LIPV, left inferior pulmonary vein

First, upper to middle mediastinal dissection was performed using a 4-cm left cervical incision under single-port (GelPOINT® Mini, Applied Medical Co., Ltd, Rancho Santa Margarita, CA, USA) inflatable mediastinoscopy (Fig. 1a). From the left neck, we dissected the upper and middle mediastinal lymph nodes, including the lymph nodes around the left recurrent laryngeal nerve, carina, and bilateral main bronchi (Fig. 2). Subsequently, a stomach tube was constructed, and dissection around the celiac axis was performed under laparoscopy (Fig. 1b). Next, transhiatal lower mediastinal dissection was performed using a mediastinoscope from the esophageal hiatus. Finally, we extended the left cervical incision to the right side (Fig. 1c) and performed cervical dissection in the direct view; 3FD was then completed. We usually selected stomach tube reconstruction with a mediastinal route, and esophago-stomach tube anastomosis was performed by a circular stapler in the neck.

### Statistical analysis

Overall survival (OS) was measured from the date of the start of chemotherapy to the date of death or last follow-up. Relapse-free survival (RFS) was measured from the date of the start of chemotherapy to the date of the first evidence of tumor recurrence. Relapse-free patients were censored at the date when the absence of relapse was confirmed. OS and RFS were calculated by the Kaplan-Meier method. All statistical analyses were performed by the freely available software EZR version 1.37 [17].

The clinical and pathological staging of the tumors were classified according to the tumor-node-metastasis classification of the Union for International Cancer Control, eighth edition [18]. Pathological response of chemotherapy was classified according to the Japanese Classification of Esophageal Cancer, eleventh edition [19]. AEs during chemotherapy were classified according to the National Cancer Institute’s Common Terminology Criteria for Adverse Events, version 5.0 [20]. Postoperative complications were described according to the Clavien-Dindo classification, version 2.0 [21].

## Results

### Study population

Nineteen patients, who underwent esophagectomy for esophageal or esophagogastoric junction carcinoma in our department between June 2015 and December 2017, were screened for this study, and 16 patients were judged to be eligible. The reasons for ineligibility were as follows: adenocarcinoma of esophagogastric junction (*N* = 2) and estimated superficial ESCC without lymph node metastasis (*N* = 1).

### Patient characteristics

Sixteen patients with ESCC were included. All patients were diagnosed as having clinical stage II to IVa disease. For these 16 patients, two cycles of DCF therapy were administered, followed by radical TME. The characteristics of the patients are summarized in Table 1.

**Table 1:** Characteristics of patients in this cohort

Parameters	*N*=16
Age	
Median (year) (Range)	71.5 (54-80)
Gender	12
Male	4
Female	
ECOG performance status	
0	15
2	1
Main location of the primary tumor	
Upper thoracic	1
Middle thoracic	12
Lower thoracic	3
Clinical TNM (UICC TNM 8th)	
T2	1
T3	15
N0	2
N1	8
N2	5
N3	1
M0	16
Clinical stage (UICC TNM 8th)	
II	3
III	12
IVA	1

*ECOG* Eastern Cooperative Oncology Group, *TNM* tumour node metastasis, *UICC* Union for International Cancer Control

### Chemotherapy and adverse events

Although 6 of 16 patients (37.5%) received a reduced dose of chemotherapy because of low-performance status, low renal function, or occurrence of AEs, all 16 patients completed two cycles of DCF therapy. Therefore, safety analysis was performed in all patients. AEs during chemotherapy are shown in Table 2. Leukopenia and neutropenia occurred in most of the patients; however, they were controllable. Other than leukopenia and neutropenia, grade ≥ 4 AEs were not observed. There was no mortality during chemotherapy.

**Table 2:** Adverse events during chemotherapy

	Grade 1	Grade 2	Grade 3	Grade 4	%Grade 3/4
Laboratory findings					
WBC decreased	5	0	9	2	68.8
Neutrophil count decreased	0	1	7	8	93.8
Anemia	7	0	0	0	0
Hypoalbuminemia	5	11	0	0	0
Total bilirubin increased	1	1	0	0	0
AST increased	1	0	0	0	0
ALT increased	2	0	0	0	0
Hyponatremia	4	0	0	0	0
Hypokalemia	2	0	0	0	0
Creatinine increased	2	0	0	0	0
Objective findings					
Febrile neutropenia	―	―	5	0	31.3
Lung infection	0	0	1	0	6.3
Nausea	4	4	0	0	0
Diarrhea	3	5	4	0	25.0
Constipation	8	2	1	0	6.3
Abdominal pain	7	0	0	0	0
Herpes simplex reactivation	0	1	0	0	0

*WBC* white blood cells, *AST* aspartate aminotransferase, *ALT* alanine transaminase

### Surgical treatment

All 16 patients were scheduled for TME. Fifteen successfully completed TME, but 1 was converted to open TTE because of intraoperative tracheal injury. 3FD was performed in 12 of 16 patients (75%), and R0 resection was achieved in 15 of 16 patients (93.8%). The median postoperative hospital stay was 16 days. Surgical findings are summarized in Table 3.

**Table 3:** Surgical findings

Parameters	*N*=16
Surgical procedure	
TME (non-transthoracic)	15
Converted to open TTE	1
Lymph node dissection	
≤ 2-field dissection	4
3-field dissection	12
Operation time	
Median (min) (Range)	489 (430-616)
Intraoperative blood loss	
Median (mL) (Range)	180 (30-665)
Residual tumor	
R0	15
R1	1
Harvested lymph nodes	
Median (Range)	57.5 (36-95)
Postoperative hospital stay	
Median (days) (Range)	16 (12-67)

*TME* transmediastinal esophagectomy, *TTE* transthoracic esophagectomy

### Operative morbidity and mortality

The median operation time was 489 min, and the median intraoperative blood loss was 180 mL. The only intraoperative complication was one case of tracheal injury. Postoperative complications are shown in Table 4. Grade IIIb chylothorax was observed in three patients, and re-operation was required in these patients. Grade IIIa recurrent nerve paralysis was observed in one patient. Anastomotic leakage was not observed; however, as a late complication after discharge, anastomotic stricture was observed in six patients. There was no surgical mortality.

**Table 4:** Postoperative complications

Complications	Clavien-Dindo classification				
	I	II	IIIa	IIIb	%IIIa/IIIb
Early complications (In hospital)					
Chylothorax	0	0	0	3	18.8
RLN paralysis	2	0	1	0	6.3
Pneumonia	0	2	0	0	0
Deep vein thrombosis	0	1	0	0	0
Late complications (After discharge)					
Anastomotic stricture	0	0	6	0	37.5

*RLN* recurrent laryngeal nerve

### Pathological findings and response to chemotherapy

The pathological findings are shown in Table 5. A complete pathological response to preoperative chemotherapy (grade 3) was observed in two patients, and partial response (grades 1b and 2) was observed in seven patients; therefore, the pathological complete response rate was 12.5% (2/16), and the pathological response rate was 56.3% (9/16).

**Table 5:** Pathological findings

Parameters	*N*=16
Pathological TNM (UICC TNM 8th)	
T0	2
T1a	1
T1b	3
T2	3
T3	8
N0	6
N1	7
N2	2
N3	1
M0	15
M1	1*
Pathological stage (UICC TNM 8th)	
0	2
IB	2
IIA	1
IIIA	2
IIIB	7
IV	2
JES-pathological response	
Grade 1a	7
Grade 1b	3
Grade 2	4
Grade 3	2

*UICC* Union for International Cancer Control, *TNM* tumour node metastasis, *JES* Japan Esophageal Society

^*^Due to intramural metastasis to the stomach

### Overall and relapse-free survival

**Fig. 3:**
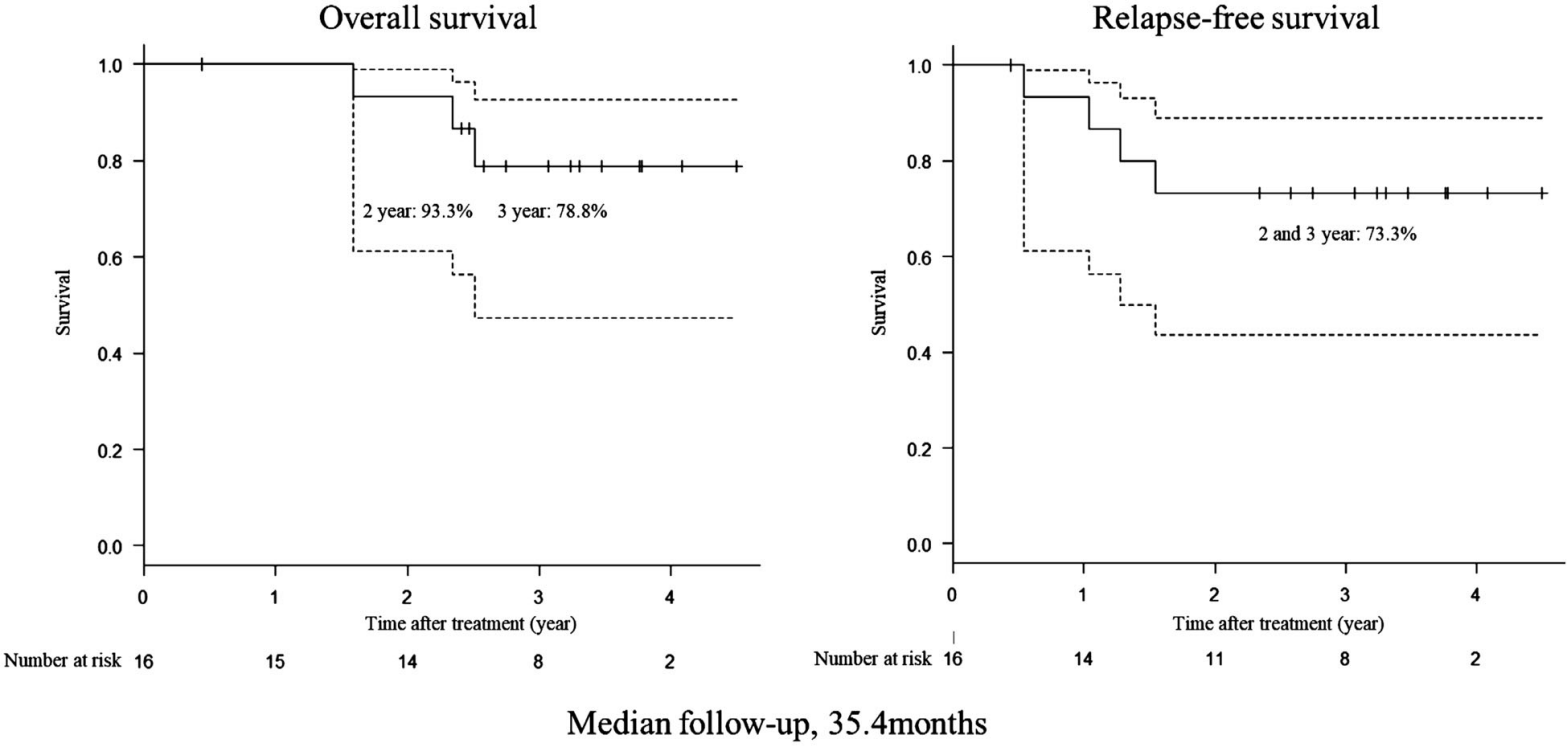
The overall survival (OS) and relapse-free survival (RFS). The median follow-up of the 16 patients was 35.4 months. The 2-year OS was 93.3% (95% CI, 61.3–99.0), and the 3-year OS was 78.8% (95% CI, 47.3–92.7). The 2-year and 3-year RFS were both 73.3% (95% CI, 43.6–89.1). CI, confidence interval

The OS and RFS curves are shown in Fig. 3. The median follow-up of the 16 patients was 35.4 months. The 2-year OS was 93.3% (95% confidence interval [CI], 61.3–99.0), and the 3-year OS was 78.8% (95% CI, 47.3–92.7). The 2-year and 3-year RFS were both 73.3% (95% CI, 43.6–89.1). Three patients died during follow-up; two died of tumor recurrence, and one died of secondary-induced acute myeloid leukemia.

## Discussion

This is the first report of long-term survival of patients undergoing TME with NAC for locally advanced ESCC. Our study demonstrated that the combination of preoperative DCF therapy and TME for thoracic ESCC could be performed safely and might lead to better prognosis than the current standard treatments. In the JCOG9907 trial, which assessed NAC for ESCC, the 3-year OS was 62%, and the 5-year OS was 55%; these are the highest survival rates observed among similar randomized controlled trials (RCTs) [2, 4, 22]. The study by Natsugoe et al., which was an RCT assessing NACRT, had a 3-year OS of 57%; this is the highest survival among RCTs assessing NACRT [2, 22, 23]. Conversely, our study showed a 3-year OS of 78.8%. Although it is difficult to compare our results with those of RCTs because our study was a retrospective observational study, our results seemed not to be so bad. There are two possible reasons for this discrepancy: (1) the superiority of DCF therapy and (2) the curability and low invasiveness of the TME procedure.

Neoadjuvant DCF therapy in our study demonstrated relatively high toxicity; however, it had a remarkable tumor control effect, with 12.5% (2/16) of patients having complete response and 56.3% (9/16) having pathological response. In the JCOG9907 trial, the complete response rate of NAC (CF therapy) was 2.4%, and the clinical response rate was 38% [4]. Therefore, triplet DCF therapy seems to be more effective than doublet CF therapy. Hara et al. reported a phase II study on preoperative DCF therapy and found good results, with 17.1% (7/41) achieving a grade 3 response and 34.1% (14/41) achieving a grade 2 response [24]. Therefore, they saw greater effectiveness of DCF than we did. However, in their study, DCF therapy consisted of docetaxel and cisplatin at 70 mg/m^2^ on day 1 and 5-FU at 750 mg/m^2^/day on days 1–5 repeated three times every 3 weeks, so their patients received a higher cumulative dose than those in our study [24]. In Natsugoe’s study, NACRT consisted of 40 Gy of radiation and CF therapy (cisplatin 7 mg over 2 h and 5-FU 350 mg over 24 h during radiotherapy), and in the NACRT arm, 13.6% (3/22) of patients had a grade 3 response and 31.8% (7/22) had a grade 2 response [23]. Therefore, we conclude that neoadjuvant triplet chemotherapy is superior to doublet chemotherapy and comparable to CRT in terms of patient response. In Japan, the JCOG1109 trial, which is a triple-arm RCT comparing CF versus DCF versus radiotherapy with CF as preoperative therapy, is now ongoing [25].

In terms of AEs, DCF therapy showed a higher rate of grade 3/4 toxicities than what has been previously observed with CF therapy or CRT, especially in hematological components (leukopenia and neutropenia) [4, 23, 24]. However, leukopenia and neutropenia were manageable, and neither our study nor Hara’s phase II study had any chemotherapy-related death [24]. Therefore, neoadjuvant triplet chemotherapy seems to be safe. Although the DCF therapy in Hara’s study had a higher dose than ours, there was no difference in the extent of AEs between these two studies [23]. Hence, we might have been able to introduce more powerful DCF therapy.

As we mentioned in the introduction, the standard treatment for locally advanced ESCC in Japan is esophagectomy with 3FD, and it is believed that 3FD requires TTE. Therefore, TTE was performed for esophagectomy in the JCOG9907 trial and Hara’s study (Natsugoe’s study also suggested that TTE was performed; however, details were not provided) [4, 23, 24]. In contrast, we performed TME with 3FD in this study. The biggest difference between TME and TTE is that TME does not injure the chest wall and pleura. TME can preserve those chest structures and therefore is associated with fewer respiratory complications than TTE [11]. Infectious complications, including respiratory complications, may be directly linked to prognosis after esophageal cancer surgery [8, 9]; this was shown in the exploratory analysis of JCOG9907 [10]. In our study, we observed only two cases of pneumonia as infectious complications, and this result might have led to a better prognosis.

Some argue that TME is less radical than TTE. Previously, it was difficult to dissect the mediastinal lymph nodes, especially the nodes around the left recurrent laryngeal nerve or carina, without a transthoracic approach. However, the development of videoscope and surgical devices has enabled mediastinal dissection without a transthoracic approach [15, 16]. We performed upper to middle mediastinal dissection under single-port inflatable mediastinoscopy from the left neck. We dissected 57.5 lymph nodes per operation and could radically dissect lymph nodes around the left recurrent laryngeal nerve, carina, and bilateral main bronchi (Fig. 2). This less invasive procedure may be beneficial to patient prognosis. In Hara’s study, the 2-year progression-free survival was 74.5%, and the 2-year OS was 88.0% [24]; in contrast, in our study, the 2-year RFS was 73.3%, and the 2-year OS was 93.3%. Even though a higher dose of DCF therapy was administered in Hara’s study [24], our study had comparable results. This might indicate the superiority and radicality of our TME procedure, despite its less invasive nature.

However, our procedure was not without complications. We experienced a case of intraoperative trachea injury. The trachea or large blood vessels are complicatedly arranged around the esophagus, and intraoperative damage to them can be fatal. Therefore, a deep understanding of mediastinal anatomy is essential for TME. We had three cases of chylothorax that required re-operation. All three cases could be controlled by laparoscopic transhiatal thoracic duct ligation, and patients recovered quickly [26]. Our TME procedure usually preserved the thoracic duct; however, highly advanced tumors would have required ligation or excision of the thoracic duct. Another complication was anastomotic stricture. We did not observe anastomotic leakage during treatment; however, six cases of anastomotic stricture requiring balloon dilation were observed after discharge. We used a circular stapler for esophagogastric tube anastomosis; it might have been possible to reduce anastomotic stricture by using a linear stapler or hand sewing.

Combined neoadjuvant DCF and radical esophagectomy under TME showed fairly good results; however, this was a single-center, retrospective study in a small number of patients. To truly demonstrate the usefulness of our treatment, it is necessary to perform RCTs to verify both the superiority of neoadjuvant DCF and the superiority of TME to TTE. Regarding neoadjuvant DCF, as we mentioned above, the JCOG1109 trial, which compares doublet chemotherapy, triplet chemotherapy, and chemoradiotherapy, is currently ongoing. Therefore, our future direction should be to promote an RCT comparing the superiority of TME to TTE for resectable locally advanced esophageal cancer.

## Conclusion

Despite some complications, our combination therapy of neoadjuvant DCF and TME for locally advanced ESCC had a satisfactory long-term prognosis with no treatment-related mortality. These results suggest that triplet DCF might be safe and more effective than doublet CF or CRT as neoadjuvant therapy, and TME might be less invasive than TTE while providing treatment that is as radical.

## Data Availability

The datasets used and analyzed during the current study are available from the corresponding author on reasonable request.
